# Nitrous Oxide

**Published:** 1870-12

**Authors:** James B. Hodgkin

**Affiliations:** Alexandria, Va.


### ARTICLE II.

### *Nitrous Oxide.*

**Review of Article in New York Medical Journal, August 1870, on Nitrous Oxide as an Anæsthetic, by R. Armory M. D of Massachusetts.**

By James B. Hodgkin, D. D. S. Alexandria, Va.

This agent has become so every day an affair in the hands of the dental surgeon, and so exclusively appropriated by the fraternity as an anæsthetic, as to seem almost to belong to us who are coarsely said to "find provison for our teeth, by pulling other people's out;" and any investigation into its physiological action by the medical profession is apt to be slighted by us who are prone to think that the daily administration of "gas" without question and without accident to all who apply for it, is sufficient evidence of its innocuousness, and is sufficient knowledge for practical use. And indeed it may seem that such knowledge is sufficient when it is considered how little value those who take gas at the hands of any one who will give it, seem to set upon their own lives by placing them in the hands of reckless and ignorant parties. To this class of practitioners is perhaps due the credit of bringing nitrous oxide prominently before the public through its indiscriminate use, though the same public is of course dependent on some one else for a candid and impartial statement of its merits, and for a philosophic investigation into the rationale of its operation as an anæsthetic.

The New York Medical Journal for August 1870, contains an article from the pen of R. Armory M. D. of Mass., in which full details are given of a systematic and philosophic investigation of anæsthesia by nitrous oxide, and the conclusions of the author as to the cause of anæsthesia by that agent, with his reasons therefor. The experiments of Dr. A. bear the appearance of close observation, and his conclusions, if not sound, are logically so from his standpoint.

He states that he "commenced his experiments with the feeling that he was concerned with a dangerous anæsthetic, which was too commonly used by dentists and quacks," but arrives at quite a different opinion at the end. It is impossible within the brief limits proposed for this paper to give anything more than a condensed statement of the main points brought out by the investigations of Dr. Amory, which were first directed towards ascertaining the amount of carbonic acid retained in and eliminated from the blood during natural respiration, and during the different stages of anæsthesia. Accuracy in these experiments are confessedly difficult, and the author does not profess to give more than a proximate result.

Observation of the phenomena induced by the inhalation of nitrous oxide having led him to the belief that they were mainly due to an accumulation of carbonic acid in the blood, a series of experiments were instituted to ascertain the amount of this last gas accumulated and eliminated during anæsthesia by nitrous oxide. A number of experiments made on a dog showed that the amount of carbonic acid eliminated from the blood during the unconscious state was reduced one half, leading to the supposition that the effects of the gas might be due as above stated to the accumulation of carbonic acid in the blood, or perhaps that oxygenation of the blood is prevented and carbonic acid, the result of oxygenation, withheld; which latter he deemed the more plausible theory from the fact that immediately after recovery from the effects of the gas the amount of carbonic acid eliminated was still small, it being necessary, he thinks, that the nitrous oxide taken into the blood be thrown off, before the carbonic acid can be produced in its normal quantity.

This accumulation of carbonic acid in the blood, is, he thinks may be an effect of the anæsthesia, not the cause.

In a number of experiments made on pigeons, one of these birds lived for forty minutes in a small receiver containing nitrous oxide, and then perished accidentally, while one confined in a smaller jar containing air, lived one hour and twenty-four minutes. This bird was in a comatose state in twenty-two minutes. Another pigeon lived thirtv-two and a rabbit fifty-three minutes in a jar containing nitrous oxide.

"Two or three times," says Dr. Armory, "it has happened to me, when I had thought an animal dead from asphyxia, after the [prolonged] inhalation of nitrous oxide, to be supprised by voluntary respiration recurring after I had removed the muzzle. In fact I have now two dogs alive who had not respired for one whole minute when undergoing an experiment. Never has an animal died unexpectedly, and it was always very difficult to cause asphyxia if the smallest modicum of air passed into the lungs."

The experimenter thinks that death in all these cases was caused by asphyxia and not by paralysis of the hearts' action, nor probably from venous congestion; "nor was it probable that so inert a substance in the blood could cause paralysis of the nerves, as the contact of ether or chloroform may do."

By experiments performed on isolated nerves it was ascertained that their conductive power [outwards] was not diminished during the anæsthetic condition.

Some interesting details are given of experiments on the cerebral circulation, which the author thinks establish the truth of his hypothesis that capillary stasis occurs in the brain; and also a number of diagrams of sphygmographic traces of the radial artery, taken before, during and after the inhalation of gas. These diagrams show that during the anæsthetic state the pulse is hurried though diminished in power. The experimenter thinks that "these experiments prove that the gas, though respirable, (that is, capable of passing in and out of the lungs,) yet would not deliver up its oxygen to the blood, nor cause the elimination of as much carbonic acid from the blood as if atmospheric air were respired. He considers "this affect upon the capillary system to be caused by the non aræation of venous blood in the lungs;" and thinks that in asphyxia, (as he is pleased to call the extreme condition of anæsthesia produced by N. O.) caused by the gas in question, "the process of respiration ceases *after* inspiration and *before* expiration," and that the first impulse of returning respiration is to *expire* the gas from the lungs. He says:

"All animals, when they appear to respire [while under the influence of N. O.] can be awakened to consciousness almost immediately. But at a certain stage they appear to stop all attempts at respiring, and lie motionless. If not forced to inhale air they will die. The gas will not then support life."

But a seeming contradiction to this is given on a subsequent page, when he says:

"I have observed that the cardiac pulsations persist for a long time after the cessation of the respiratory function, and that a rabbit supposed to be dead fifteen minutes before, was picked up and thrown upon the table before proceeding to an autopsy, and immediately began to respire and lived several days, until the exigencies of a subsequent experiment demanded the sacrifice of its life."

Arriving at the conclusion from his experiments that "capillary stasis" is the cause of anæsthesia by nitrous oxide, Dr. Armory says he does not think death from it due to syncope, though he quotes Prof. Brown-Sequard as authority that this cause may produce death. He says:

"The capillary stasis is only transient and seems to depend on defective respiration. "***The anæsthesia is caused by insufficient oxydation of the tissues by means of the blood. If the lungs be forced to receive this gas, they respire it without decomposing the oxygen, and death results from asphyxia; or if air be allowed, the animal revives. ** Generally there is a period of three or four minutes after inspiring air that loss of sensation persists. If the gas be re-applied before the expiration of this period the loss of sensation may be kept up for a longer time."

The blood, owing to an insufficient supply of respirable oxygen, accumulates the pre-existing amount of carbonic acid in the blood, and in this way causes an arrest of capillary circulation."

Prof. Geo. Johnson is quoted as stating that there is no reason to suppose the inhalation of either nitrous oxide or nitrogen causes an accumulation of carbonic acid in the blood. Dr. Armory states that while "during anæsthesia from nitrous oxide the amount of carbonic acid eliminated was two thirds of that during consciousness, immediately after the inhalation and after recovery from the anæsthetic condition, the carbonic acid thrown off was only *one third the amount exhaled during anæsthesia*;" and he thinks that during that state "the free carbonic acid in the lungs is given off and until the stagnation in the capillary circulation is attained there is only a modification of combustion. When this capillary stasis occurs, there is no combustion, and temporary death to the nerve substance is effected. On the inspiration of air the combustion is renewed, and the product of oxygenation or combustion, viz: carbonic acid, does not *immediately* appear in the expired air. The nitrous oxide must be eliminated first. ***. There is no poisonous substance in the blood. There is simply arrest of capillary circulation."

*To be Continued.*

